# Cardiovascular cine imaging and flow evaluation using Fast Interrupted Steady-State (FISS) magnetic resonance

**DOI:** 10.1186/s12968-018-0433-3

**Published:** 2018-02-19

**Authors:** Robert R. Edelman, Ali Serhal, Amit Pursnani, Jianing Pang, Ioannis Koktzoglou

**Affiliations:** 1 Radiology, Northshore University HealthSystem, Evanston, IL USA; 2 Radiology, Northwestern Memorial Hospital, Chicago, IL USA; 3 Medicine, Northshore University HealthSystem, Evanston, IL USA; 4 Medicine, University of Chicago Pritzker School of Medicine, Chicago, IL USA; 5 Siemens Medical Solutions USA Inc., Chicago, IL USA; 6 Radiology, University of Chicago Pritzker School of Medicine, Chicago, IL USA; 7 Evanston, IL USA

**Keywords:** Cine, Coronary arteries, Magnetic resonance imaging, Flow measurement, Angiography, Quiescent interval slice-selective, Fast interrupted steady-state

## Abstract

**Background:**

Existing cine imaging techniques rely on balanced steady-state free precession (bSSFP) or spoiled gradient-echo readouts, each of which has limitations. For instance, with bSSFP, artifacts occur from rapid through-plane flow and off-resonance effects. We hypothesized that a prototype cine technique, *radial fast interrupted steady-state (FISS)*, could overcome these limitations. The technique was compared with standard cine bSSFP for cardiac function, coronary artery conspicuity, and aortic valve morphology. Given its advantageous properties, we further hypothesized that the cine FISS technique, in combination with arterial spin labeling (ASL), could provide an alternative to phase contrast for visualizing in-plane flow patterns within the aorta and branch vessels.

**Main body:**

The study was IRB-approved and subjects provided consent. Breath-hold cine FISS and bSSFP were acquired using similar imaging parameters. There was no significant difference in biplane left ventricular ejection fraction or cardiac image quality between the two techniques. Compared with cine bSSFP, cine FISS demonstrated a marked decrease in fat signal which improved conspicuity of the coronary arteries, while suppression of through-plane flow artifact on thin-slice cine FISS images improved visualization of the aortic valve. Banding artifacts in the subcutaneous tissues were reduced. In healthy subjects, dynamic flow patterns were well visualized in the aorta, coronary and renal arteries using cine FISS ASL, even when the slice was substantially thicker than the vessel diameter.

**Conclusion:**

Cine FISS demonstrates several benefits for cardiovascular imaging compared with cine bSSFP, including better suppression of fat signal and reduced artifacts from through-plane flow and off-resonance effects. The main drawback is a slight (~ 20%) decrease in temporal resolution. In addition, preliminary results suggest that cine FISS ASL provides a potential alternative to phase contrast techniques for in-plane flow quantification, while enabling an efficient, visually-appealing, semi-projective display of blood flow patterns throughout the course of an artery and its branches.

**Electronic supplementary material:**

The online version of this article (10.1186/s12968-018-0433-3) contains supplementary material, which is available to authorized users.

## Background

Existing cine imaging techniques for the cardiovascular system rely on balanced steady-state free precession (bSSFP) or spoiled gradient-echo (sGRE) readouts, each of which has significant limitations. For instance, fat appears bright with cine bSSFP. As a result, small-caliber embedded structures such as the coronary and internal mammary arteries are obscured by artifacts from fat/water chemical shift. Cine bSSFP is also susceptible to artifact from rapid through-plane flow and banding artifact from off-resonance effects. Alternatively, cine sGRE suffers from inferior signal-to-noise ratio and temporal resolution, as well as considerable in-plane flow saturation.

We hypothesized that a prototype cine imaging technique, *radial fast interrupted steady-state (FISS)* [1], could overcome these limitations. The technique was compared to standard Cartesian cine bSSFP in a small group of healthy subjects to evaluate cardiac function, coronary artery conspicuity, and aortic valve morphology. Given its advantageous properties, we further hypothesized that the cine FISS technique, in combination with arterial spin labeling (ASL), could provide an alternative to phase contrast for visualizing and quantifying in-plane flow within the aorta and branch vessels.

## Methods

The study was IRB-approved, and subjects provided written informed consent. Eight healthy subjects (5 male, age = 24 to 54 years) were imaged at 1.5 T (MAGNETOM Avanto, Siemens Healthineers, Erlangen, Germany). Following standard localizer scans, breath-hold cine images were acquired in the left ventricular (LV) 2-chamber, 3-chamber, and 4-chamber views, as well as obliquely through the aortic valve.

### Cine FISS

FISS differs from conventional bSSFP in that it disrupts the steady-state magnetization at frequent intervals. The steady-state magnetization undergoes gradient and radiofrequency (RF) spoiling after each block of bSSFP modules (5 to 8 in this study) to suppress off-resonant and out-of-slice spins. To avoid artifacts that would otherwise occur from these repeated disruptions, the technique uses a radial k-space trajectory with equidistant view angles.

To evaluate the relative benefits and limitations of this new technique in the heart, breath-hold cine FISS and Cartesian cine bSSFP were acquired using identical spatial resolution, numbers of shots and cine frames. Retrospective electrocardiographic (ECG)-gated cine imaging was performed with standard inline reconstruction of 32 cine frames. Scan parameters included: scan time = 12 heart beats per slice, 96 radial views for cine FISS, ipat factor = 2 for Cartesian cine bSSFP, acquisition matrix = 144, field of view = 340-mm, 12 shots, sampling bandwidth = 1085 Hz/pixel, echo time ~ 1.3 msec, sequence repetition time ~ 2.6 msec, flip angle ~ 70 degrees. Slice thickness was 6-mm. For imaging of the aortic valve, scans were repeated using 2-mm slices, and radial cine bSSFP was acquired in addition to Cartesian cine bSSFP and cine FISS.

Image evaluation was performed by a radiologist with training in noninvasive cardiac imaging. *Quantitative analysis:* Biplane LV ejection fraction was calculated (cvi42, Circle Cardiovascular Imaging, Calgary, Canada) using the 2-chamber and 4-chamber long axis views. Epicardial and subcutaneous fat to right ventricular (RV) blood pool contrast-to-noise ratios (CNR) were calculated as: (signal_blood pool_ - signal_fat_) / temporal standard deviation of signal in nearby hypointense lung tissue.

*Qualitative analysis:* Cine image quality for the heart was graded using a 4-point scale (1 =LV myocardium not visualized, severe artifact; 2 = myocardium poorly visualized; moderate artifact; 3 = myocardium moderately well visualized, mild artifact; 4 = myocardium well visualized, negligible artifact.) Conspicuity of the aortic valve at peak systole was rated on a 4-point scale ranging from 1 = aortic valve leaflets not visualized, severe artifact to 4 = aortic valve leaflets well visualized, negligible artifact. Coronary artery conspicuity was rated on a four-point scale, ranging from 1 = left anterior descending coronary artery (LAD) not visualized, severe artifact to 4 = LAD well visualized, negligible artifact. Statistical analyses were done in SPSS (version 17.0, International Business Machines, Armonk, New York, USA). Continuous data was analyzed using paired t-tests or linear regression analysis, while ordinal data for two and three groups were compared using Wilcoxon signed-rank and Friedman tests, respectively.

### Cine FISS ASL

For localization of the aorta and branch vessels prior to flow imaging, breath-hold images were acquired with a radial quiescent interval slice-selective (QISS) pulse sequence (2-mm thick slices, 1 or 2 shots) [2]. Cine ASL using a FISS readout was used to dynamically visualize in-plane blood flow in the descending thoracic aorta and to depict flow patterns in two widely-separated aortic branch vessels (coronary and renal arteries). Spin labeling was accomplished by applying a 16 to 25-mm thick adiabatic inversion RF pulse to inflowing arterial blood. Background suppression was obtained by complex subtraction of the labeled and unlabeled cine image series, which were acquired on alternate RR intervals. Imaging parameters included 110 radial views, scan time of 16 heart beats per slice, 8 shots, and 32 reconstructed cine frames. Temporal resolution was ≈20–44 msec depending on the heart rate and number of number of bSSFP modules per block. A slice thickness of 6-mm was typically used for flow quantification in the aorta and renal arteries. In addition, slice thicknesses up to 48-mm were tested for semi-projective imaging, with the goal of displaying the entire length and thickness of a target vessel in a single cine image series.

For the coronary arteries, the QISS image showing the longest length of the LAD coronary artery was used to center a five-slice (overlap = 20%, one slice per breath-hold) cine FISS ASL acquisition using 3-mm thick slices. Cine ASL imaging of the right coronary artery (RCA) was not included due to time limitations.

#### Flow phantom

A pulsatile flow circuit consisting of 6.35 mm diameter tubing filled with a 70% water/30% glycerin mixture (pumping frequency 60 Hz) was used to validate the cine FISS ASL measurement of flow velocity, as given by the ratio of: (mean distance traveled by the tagged bolus over one pump cycle) / (pump cycle duration).

#### In-plane flow velocity quantification

For the aorta, maximal flow velocity was quantified as the ratio of: (distance traveled by the leading edge of the tagged blood at peak systole) / (frame duration). Breath-hold 2D cine phase contrast with a through-plane velocity encoding of 150 cm/s was used as the reference standard. Given the small caliber of the coronary arteries, maximum intensity projections of several thin overlapping cine FISS ASL slices for each diastolic frame were analyzed to ensure that the labeled bolus could be tracked over a sufficient vessel length.

## Results

**Fig. 1:**
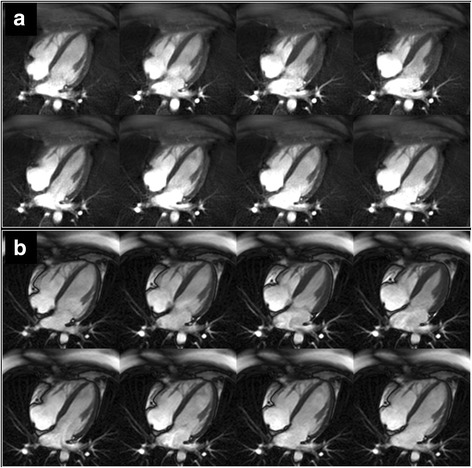
Comparison of four-chamber cine imaging of the heart (eight frames shown out of 32 acquired) using **a** cine FISS and **b** cine bSSFP. There is much better fat suppression with cine FISS as well as reduced banding artifacts in the subcutaneous tissues, but the depiction of the cardiac chambers is similar with the two techniques

The RR intervals during the CMR examinations ranged from approximately 685 msec to 1225 msec. There was no significant difference between Cartesian cine bSSFP and cine FISS in the calculated biplane LV ejection fraction (67.5% ± 4.3% vs. 68.3% ± 3.6%, p = NS) nor in qualitative image ratings for the heart (4.0 ± 0.0 vs. 4.0 ± 0.0, p = NS). Cine FISS showed much greater suppression of epicardial fat signal in all subjects, as well as reduced signal from subcutaneous fat (RV-to-epicardial fat blood pool CNR = 40.6 ± 11.4 (mean ± standard deviation) for cine FISS vs 12.7 ± 10.5 for cine bSSFP, *p* = 0.002; RV-to-subcutaneous fat blood pool CNR = 42.5 ± 10.8 for cine FISS vs 0.7 ± 8.4 for cine bSSFP, *p* < 0.001). Banding artifacts in the subcutaneous tissues were consistently less apparent with cine FISS compared with cine bSSFP (Fig. 1; see Additional file 1: Figure S1).

**Fig. 2:**
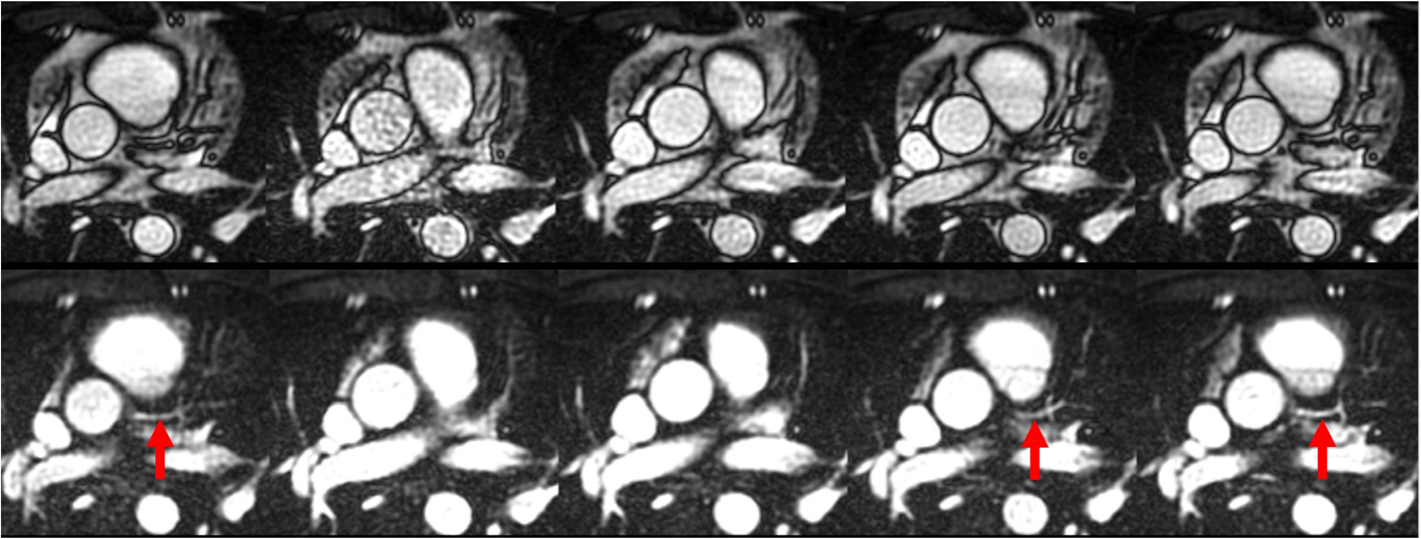
Cine imaging of the left main and left anterior descending coronary artery (LAD) coronary arteries (five frames shown out of 20 acquired) in a healthy subject. Cine bSSFP (top) fails to distinctly show the coronary arteries, whereas the LAD is well delineated (arrows) with cine FISS (bottom) due to improved epicardial fat suppression. Improved visualization of the internal mammary vessels with cine FISS is apparent as well

Compared with cine bSSFP, cine FISS significantly improved visualization of the coronary arteries (coronary artery conspicuity = 4.0 ± 0.0 for cine FISS vs. 2.6 ± 0.5 for cine bSSFP, *p* = 0.019) (Fig. 2; see Additional file 1: Figure S2).

**Fig. 3:**
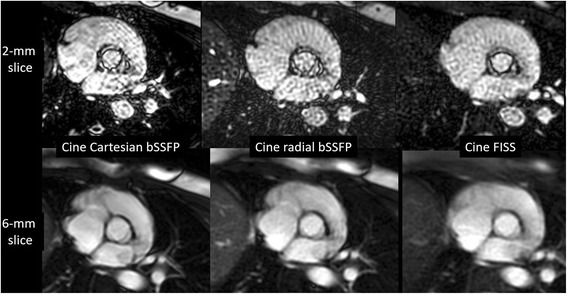
Cine imaging of the aortic valve at peak systole using Cartesian bSSFP, radial bSSFP and FISS. Image quality is similar for the three techniques using 6-mm slices (bottom row). However, with 2-mm slices (top row) image quality and conspicuity of the valve leaflets is best with cine FISS

For imaging of the aortic valve using a 6-mm slice thickness, image quality was not significantly different for cine FISS, Cartesian and radial cine bSSFP (4.0 ± 0.0 vs. 3.5 ± 0.5 vs. vs. 3.6 ± 0.5, respectively, p = NS). Conspicuity of the aortic valve leaflets was maximized by imaging with 2-mm thick slices using cine FISS, whereas thin-slice imaging resulted in increased artifacts using either Cartesian or radial cine bSSFP (Fig. 3; see Additional file 1: Figure S3). Aortic valve conspicuity values were 4.0 ± 0.0 for cine FISS versus 2.3 ± 0.5 for radial cine bSSFP and 2.4 ± 0.7 for Cartesian cine bSSFP (*p* = 0.001).

**Fig. 4:**
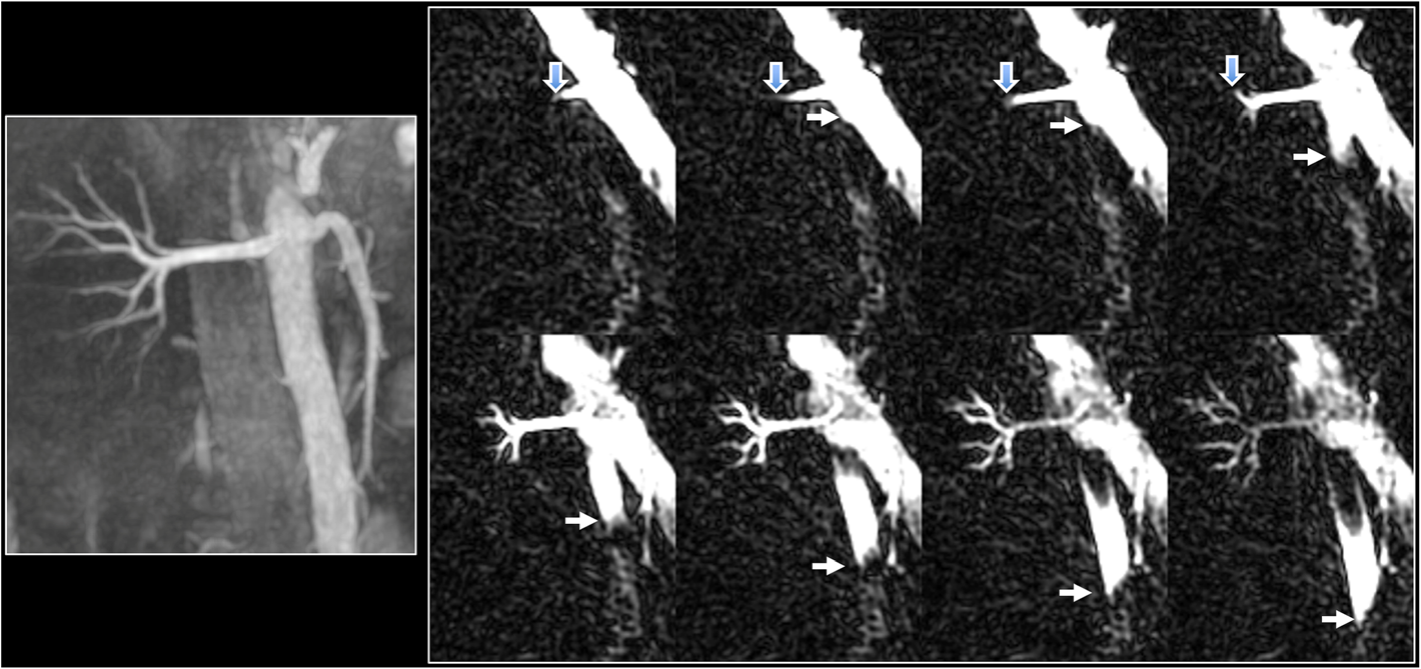
Example of cine FISS ASL for dynamic flow visualization in the right renal artery and abdominal aorta of a healthy subject. Left: maximal intensity projection (MIP) from breath-hold radial QISS acquisition. Right: Oblique coronal cine FISS ASL (eight frames shown out of 32 acquired) using 6-mm slice thickness demonstrates progression of the labeled bolus along the entire main segment of the right renal artery (open arrows) into the small intrarenal branches, as well as progression of the labeled bolus in the aorta (solid arrows)

**Fig. 5:**
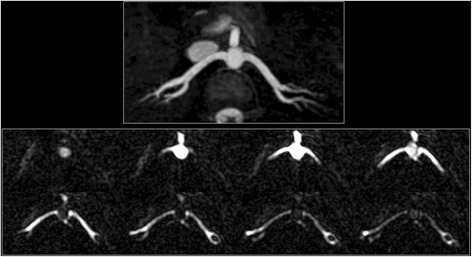
Semi-projective cine FISS ASL of the renal arteries. Top: 24-mm MIP from breath-hold radial QISS acquisition. Bottom: Axial cine FISS ASL (eight frames shown out of 32 acquired) using 24-mm slice thickness demonstrates symmetrical progression of the labeled bolus through the right and left renal arteries into the intrarenal branches

**Fig. 6:**
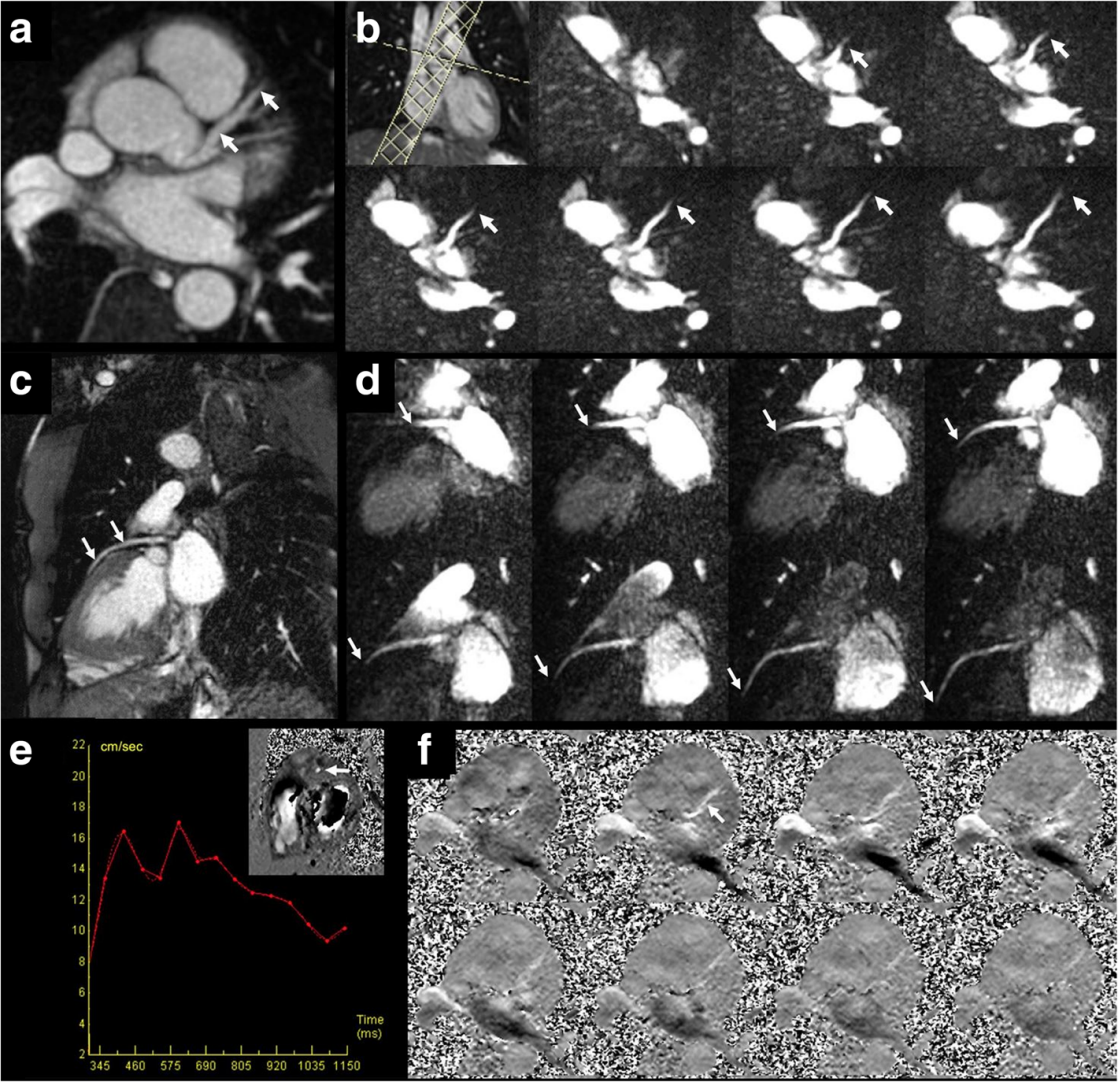
Example of cine FISS ASL for visualization and quantification of flow velocity in the left coronary arteries of a healthy subject. **a** 3-mm thick maximum intensity projection from oblique axial QISS MRA shows the left main and LAD coronary arteries (arrows). **b** Upper left frame shows graphical positioning of a 25-mm thick adiabatic inversion RF pulse through the aortic root which overlaps the sinuses of Valsalva and the ostium of the left main coronary artery. Remainder of frames (seven out of 32 acquired) acquired with cine FISS ASL (oblique axial orientation) show the progression of the labeled bolus (arrows) through the left main and LAD coronary arteries. The time between successive frames was 37.1 msec and the mean flow velocity between 444 msec and 615 msec after the R wave was 16.4 cm/s. **c** QISS MRA of the LAD (arrows) in an oblique coronal view. **d** MIP of three overlapping cine FISS ASL slices acquired in an oblique coronal plane through the LAD (eight frames displayed of 32 acquired). Blood in the aortic root was labeled in diastole, 580 msec after the R-wave (upper left frame). The labeled bolus (arrows) can be visualized as it progresses through nearly the entire length of the LAD. **e** The proximal LAD (inset, arrow) is seen in cross-section with 2D phase contrast using through-plane flow encoding (30 cm/s), permitting quantification of the diastolic flow velocity (which was similar to that measured by cine FISS ASL). **f** 2D phase contrast scan acquired with right-to-left flow encoding along the length of the LAD shows only faint, incomplete visualization of the vessel due to partial volume averaging and in-plane flow saturation

Dynamic flow patterns were well shown in the aorta, coronary and renal arteries using cine FISS ASL (Figs. 4, 5 and 6; see Additional file 1: Figure S4 and S5). The labeled bolus could be reliably visualized in subtracted images over the entire cardiac cycle as it traversed the length of the vessel. In contrast, the bolus could only be reliably visualized over a few cine frames in non-subtracted images (see Additional file 1: Figure S6).

**Fig. 7:**
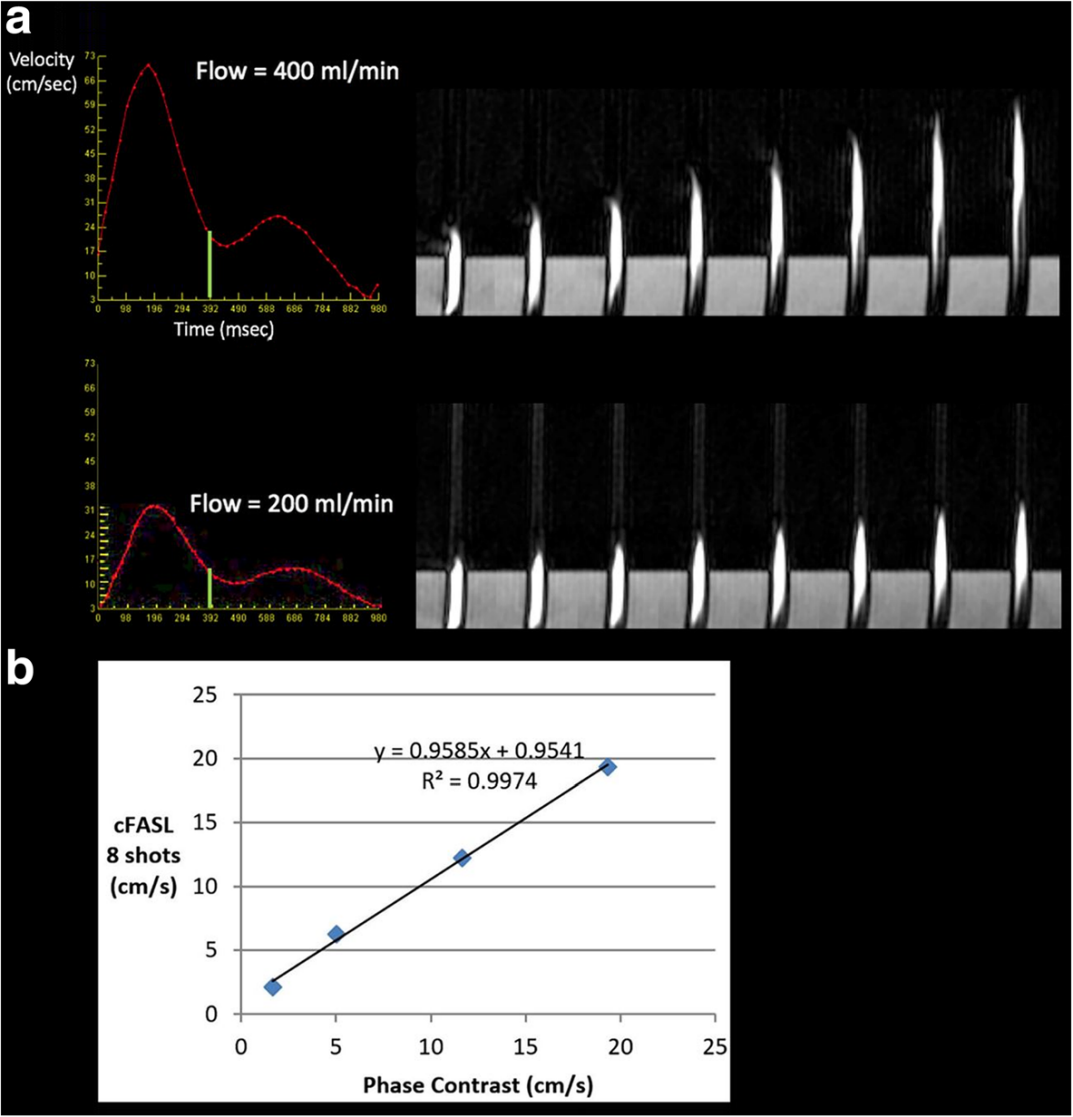
Pulsatile flow phantom with flow velocities in the expected range for the coronary arteries. **a** Illustration of cineangiographic bolus tracking (eight of 32 frames shown) using cine FISS ASL at two different flow rates (200 ml/min, 400 ml/min). The frame-to-frame displacement of the labeled bolus is directly proportional to the velocity. The vertical green line indicates the time delay following the trigger pulse when the labeling RF pulse was applied. **b** Comparison of flow measurements for 2D phase contrast and cine FISS ASL. There is excellent correlation (r^2^ = 0.9974) between the measurements

Flow velocity measurements in the pulsatile flow phantom showed excellent correlation (r^2^ = 0.997, *p* = 0.001) between cine FISS ASL and 2D cine phase contrast (Fig. 7; see Additional file 1: Figure S7).

In healthy subjects, there was excellent correlation between maximal aortic flow velocities measured by cine FISS ASL and 2D phase contrast (r^2^ = 0.959, *p* < 0.001). Mean coronary flow velocity, measured with cine FISS ASL over a ≈ 209 ± 97 msec (mean ± sd) span of diastole was 11.7 ± 3.0 cm/s. The cine FISS ASL contrast-to-noise ratio between the coronary artery and background was 16.5 ± 6.1.

## Discussion

FISS is a recently described steady-state imaging technique that has several potential advantages for cine imaging of the cardiovascular system, including: (1) pronounced suppression of fat signal; (2) elimination of through-plane flow artifacts; and (3) reduction in banding artifacts caused by off-resonance effects.

Conventional fat suppression techniques are not useful for cine bSSFP, since repeatedly interrupting the steady-state signal to apply a chemical shift-selective RF pulse can produce severe ghosting artifacts [3]. A more promising approach for fat-suppressed cine imaging is to use a periodically interrupted steady-state pulse sequence like S5FP [4] or FISS [1]. These imaging techniques demonstrate similar signal to bSSFP for on-resonant spins, but wider notches of suppression for off-resonant spins. The wider notches result in improved fat suppression, reduced banding artifact and decreased signal from off-resonant tissues located at the edges of the field of view caused by imperfect shimming.

With cine FISS, coronary artery conspicuity is greatly improved throughout the cardiac cycle because the suppression of fat signal negates chemical shift artifact at the boundary between the vessel wall and surrounding epicardial fat. The ability to rapidly image the coronary arteries throughout the cardiac cycle might add diagnostic value to conventional static navigator-gated 3D coronary CMR angiography [5] in certain cases. For instance, it might be used to dynamically asses the severity of kinking at the origin of a potentially malignant coronary anomaly, or to demonstrate phasic narrowing of a coronary bridge.

Cine bSSFP is prone to artifacts from rapid through-plane flow, which can be attributed to: (1) failure of rapidly flowing spins to attain a steady-state in the brief interval they reside within the thin slice; and (2) mislocalized signal due to steady-state magnetization persisting after the flowing spins have left the slice [6, 7]. For cross-sectional imaging of the aortic valve, we found that bSSFP flow artifacts were negligible for 6-mm thick slices but became significant when the slice thickness was reduced to 2-mm, resulting in decreased conspicuity of the valve leaflets. In contrast, the aortic valve leaflets were sharply delineated using thin-slice cine FISS. While such thin slices are not routinely used for cardiac imaging, they might be helpful in situations where high spatial resolution is needed, e.g. for precise multi-phase CMR measurements of the aortic valve apparatus in patients who are scheduled for transcatheter aortic valve replacement (TAVR) [8].

Flow imaging in the cardiovascular system is currently performed using phase contrast MRI [9]. Although 4D approaches are under active investigation, breath-hold 2D cine phase contrast is the mainstay of clinical practice and allows rapid, through-plane flow quantification in the heart and great vessels. A mechanistically-distinct alternative approach for flow imaging involves the use of ASL [10], which provides a useful quantitative tool for measuring cerebral blood flow and other perfusion indices without the need for contrast infusion [11]. In addition, ASL techniques can be used to evaluate arterial flow patterns, particularly when a cine readout is incorporated [12]. In recent years, most research efforts using cine ASL have focused on the brain, e.g. to evaluate collateral flow in the circle of Willis or flow dynamics in arterio-venous malformations [13–15]. However, cine ASL has not to our knowledge been used to measure arterial flow velocities.

The basic principle of flow velocity measurement using cine FISS ASL is fundamentally different from phase contrast. Whereas phase contrast measurements depend on flow-induced phase shifts, cine FISS ASL relies on the frame-to-frame bulk displacement of labeled spins, which translates in a straightforward way into in-plane flow velocity. Unlike phase contrast, flow velocity measurements with cine FISS ASL are relatively free from partial volume effects – a benefit of the extreme level of background signal suppression and resistance to off-resonance effects. Moreover, they are largely unaffected by gradient-induced eddy currents or Maxwell field effects that are sources of measurement error with phase contrast [16]. In principle, accurate in-plane flow velocity measurements are obtainable so long as temporal resolution and arterial conspicuity are sufficiently high. In the current study, high radial undersampling factors were used to achieve cine frame rates as high as 50 Hz in a single breath-hold acquisition. In-plane cine FISS ASL flow velocity measurements correlated well with through-plane phase contrast measurements in a pulsatile flow phantom and the aorta. Moreover, the use of a thick slice (up to 48-mm in our study) with cine FISS ASL allowed semi-projective imaging of blood flow, which is not possible with 2D cine phase contrast techniques due to partial volume averaging of flow-induced phase shifts in the artery with background phase shifts in static tissues. Using cine FISS ASL, one can dynamically visualize blood flow along the entire length of both renal arteries including intra-renal branches with a single thick-slice breath-hold acquisition (Fig. 5; see Additional file 1: Figure S5). This allows direct comparison of flow patterns and velocities in the left and right renal arteries, which might be useful for determining the hemodynamic significance of a renal artery stenosis. In addition to renal artery stenosis, the semi-projective approach has potential clinical utility in a variety of other vascular disorders. For instance, using cine FISS ASL one could rapidly evaluate flow patterns in the pulmonary arteries (see Additional file 1: Figure S8) to identify occluded branches in a patient with suspected pulmonary embolism.

Our initial empirical experience with semi-projective imaging suggests that using an excitation RF pulse with a large time-bandwidth product [17] is key to preserving arterial detail in thick-slice acquisitions, presumably because doing so maximizes the slice-select gradient and helps to overcome intravoxel dephasing from local field inhomogeneities. Alternatively, one can create a maximum intensity projection from several overlapping thin-slice cine ASL acquisitions, although this approach is less efficient and risks misregistration artifact.

### Limitations

Cine FISS has potential limitations that require further study and technical optimization. For instance, fat suppression will only be effective over a range of field strength-dependent sequence repetition times [1], which in turn may restrict the choice of certain imaging parameters such as readout bandwidth. The temporal resolution for cine FISS in our study was approximately 20% less than cine bSSFP using identical imaging parameters. This is probably not a significant limitation for most clinical applications, but the number of shots can be increased if higher temporal resolution is desired. Power deposition is slightly increased compared with bSSFP, which may limit the flip angle especially at 3 Tesla or higher field strengths.

For in-plane flow quantification with cine FISS ASL, temporal resolution must be sufficient to capture the frame-to-frame bolus transit and the labeled bolus must remain within the slice sufficiently long to be imaged over sequential cine frames. This may be problematic when only a short length of vessel is visible, particularly during rapid systolic flow. To increase temporal resolution, we have started to explore the use of a “self-subtractive” cine FISS ASL technique, which doubles temporal resolution by eliminating the unlabeled control scan normally used for image subtraction. Instead, we use a late diastolic cine frame as a mask which is subtracted from the other cine frames. Further study is needed to determine the benefits and limitations of this approach. Iterative reconstruction techniques such as compressed sensing will be helpful in permitting the use of higher acceleration factors [18]. Also, while cine FISS ASL appears well-suited for measuring velocity at selected phases of the cardiac cycle, phase contrast will be more efficient for flow volume measurements which require velocity quantification throughout the cardiac cycle.

## Conclusion

In conclusion, cine FISS demonstrates several benefits for cardiovascular imaging compared with cine bSSFP, including better suppression of fat signal and reduced artifacts from through-plane flow and off-resonance effects. The main drawback is a slight (~ 20%) decrease in temporal resolution. In addition, preliminary results suggest that cine FISS ASL provides a potential alternative to phase contrast techniques for in-plane flow quantification, while enabling an efficient, visually-appealing, semi-projective display of blood flow patterns throughout the course of an artery and its branches.

## Additional file

Additional file 1: **Figure S1.** Four-chamber cine images using Cartesian bSSFP and FISS readouts provide similar depiction of cardiac morphology and function. **Figure S2.** There is marked improvement in the degree of fat suppression using cine FISS vs. cine bSSFP, resulting in better visualization of the coronary artery (thick arrow) and the internal mammary arteries and veins (thin arrows). **Figure S3.** Cine FISS better delineates the aortic valve leaflets during systole than radial cine bSSFP or cine sGRE. In addition, cine sGRE shows signal loss due to flow saturation effects. **Figure S4.** Oblique coronal cine FISS ASL shows progression of the labeled bolus through the main segment and branches of the right renal artery. **Figure S5.** Semiprojective cine FISS ASL acquired with a 24-mm thick axial slice shows symmetrical progression of the labeled bolus through the right and left renal arteries. **Figure S6.** Dynamic imaging of blood flow in the LAD. (A) Radial QISS localizer for cine FISS ASL. (B) A 25-mm adiabatic inversion RF pulse was positioned over the aortic root and left sinus of Valsalva for spin labeling. Using cine FISS without image subtraction, the labeled bolus can only be distinctly seen in a few frames. (C and D) Cine FISS ASL with image subtraction shows the progression of the labeled through the length of the LAD over the entire duration of the cardiac cycle. **Figure S7.** Phantom study showing different rates of bolus motion using cine FISS ASL for flow rates of 200 ml/min and 400 ml/min. **Figure S8.** Cine FISS ASL of the pulmonary arteries in two different subjects. The labeling RF pulse was positioned over the right ventricle, which allowed the pulmonary arteries to be selectively displayed with only minimal signal contamination from other vessels. (PPTX 10644 kb)

## Abbreviations

ASL: Arterial spin labeling
bSSFP: balanced steady-state free precession
cine FISS ASL: cine FISS with the addition of arterial spin labeling
CMR: Cardiovascular magnetic resonance
CNR: Contrast-to-noise ratio
ECG: Electrocardiogram
FISS: Fast interrupted steady-state
LAD: Left anterior descending coronary artery
LV: Left ventricle/left ventricular
MIP: maximum image projection
QISS: Quiescent interval slice-selective
RCA: Right coronary artery
RF: Radiofrequency
RV: Right ventricle/right ventricular
sGRE: Spoiled gradient-echo
