# Supplementary material for: Cardiovascular cine imaging and flow evaluation using Fast Interrupted Steady-State (FISS) magnetic resonance
Source: J Cardiovasc Magn Reson. 2018 Feb 19;20:12. doi: 10.1186/s12968-018-0433-3 (PMC5819298; doi:10.1186/s12968-018-0433-3)
Supplement: Supplementary file 1 — Figure S1. Four-chamber cine images using Cartesian bSSFP and FISS readouts provide similar depiction of cardiac morphology and function. Figure S2. There is marked improvement in the degree of fat suppression using cine FISS vs. cine bSSFP, resulting in better visualization of the coronary artery (thick arrow) and the internal mammary arteries and veins (thin arrows). Figure S3. Cine FISS better delineates the aortic valve leaflets during systole than radial cine bSSFP or cine sGRE. In addition, cine sGRE shows signal loss due to flow saturation effects. Figure S4. Oblique coronal cine FISS ASL shows progression of the labeled bolus through the main segment and branches of the right renal artery. Figure S5. Semiprojective cine FISS ASL acquired with a 24-mm thick axial slice shows symmetrical progression of the labeled bolus through the right and left renal arteries. Figure S6. Dynamic imaging of blood flow in the LAD. (A) Radial QISS localizer for cine FISS ASL. (B) A 25-mm adiabatic inversion RF pulse was positioned over the aortic root and left sinus of Valsalva for spin labeling. Using cine FISS without image subtraction, the labeled bolus can only be distinctly seen in a few frames. (C and D) Cine FISS ASL with image subtraction shows the progression of the labeled through the length of the LAD over the entire duration of the cardiac cycle. Figure S7. Phantom study showing different rates of bolus motion using cine FISS ASL for flow rates of 200 ml/min and 400 ml/min. Figure S8. Cine FISS ASL of the pulmonary arteries in two different subjects. The labeling RF pulse was positioned over the right ventricle, which allowed the pulmonary arteries to be selectively displayed with only minimal signal contamination from other vessels. (PPTX 10644 kb) [file 12968_2018_433_MOESM1_ESM.pptx]

## Slide 1
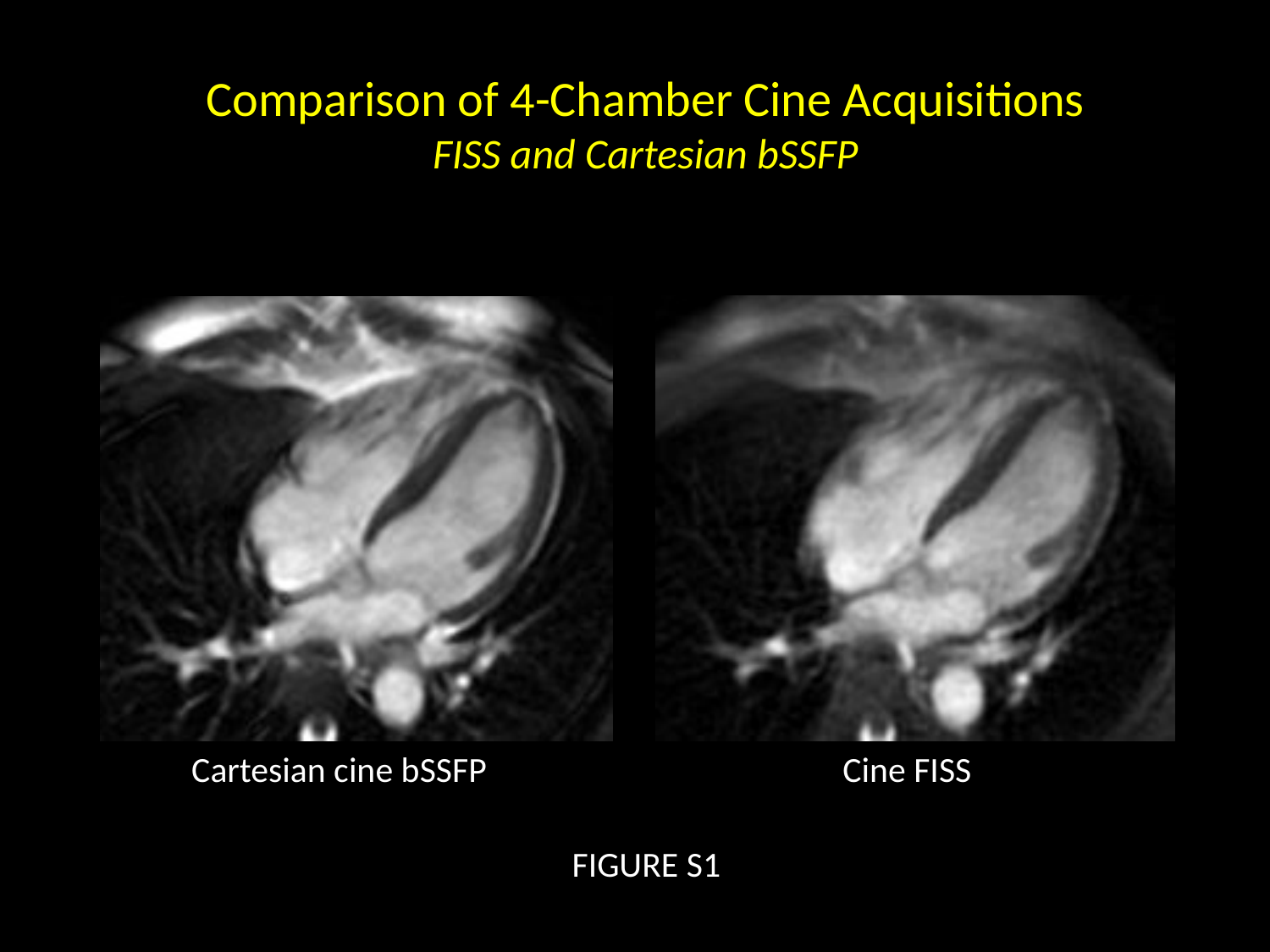

Comparison of 4-Chamber Cine Acquisitions
FISS and Cartesian bSSFP
Cartesian cine bSSFP
Cine FISS
FIGURE S1

## Slide 2
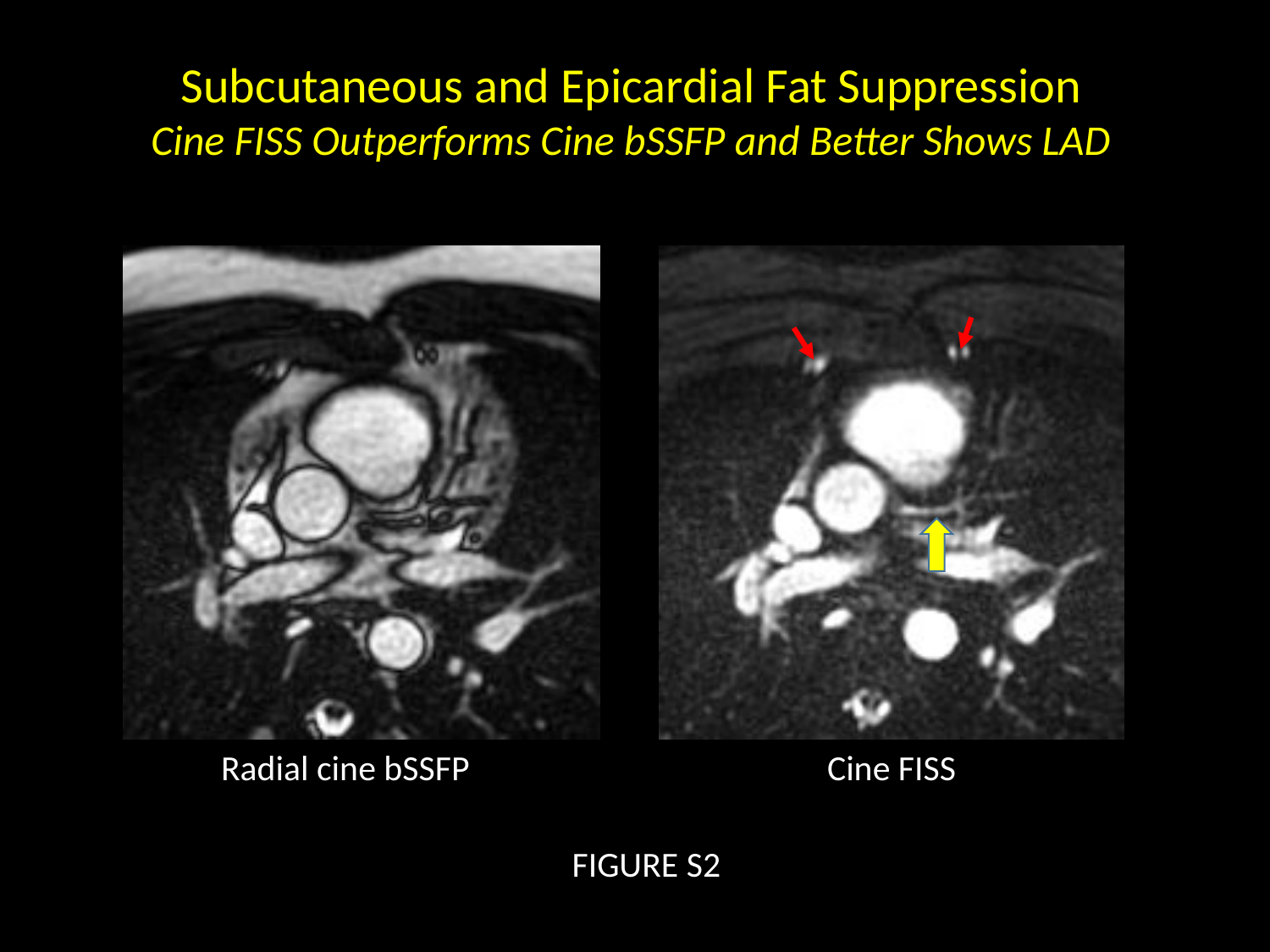

Subcutaneous and Epicardial Fat Suppression
Cine FISS Outperforms Cine bSSFP and Better Shows LAD
Radial cine bSSFP
Cine FISS
FIGURE S2

## Slide 3
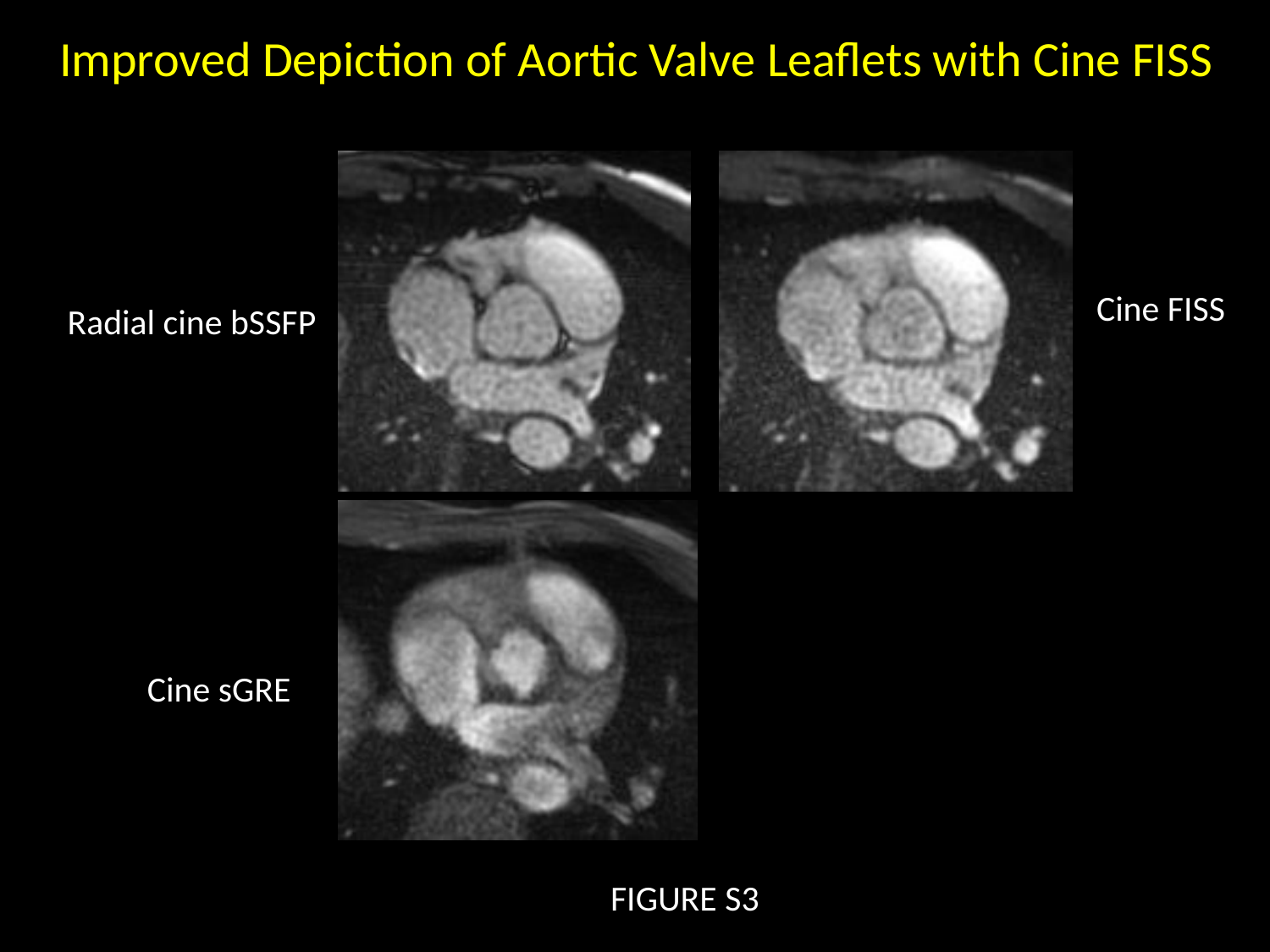

Improved Depiction of Aortic Valve Leaflets with Cine FISS
Cine FISS
Radial cine bSSFP
Cine sGRE
FIGURE S3

## Slide 4
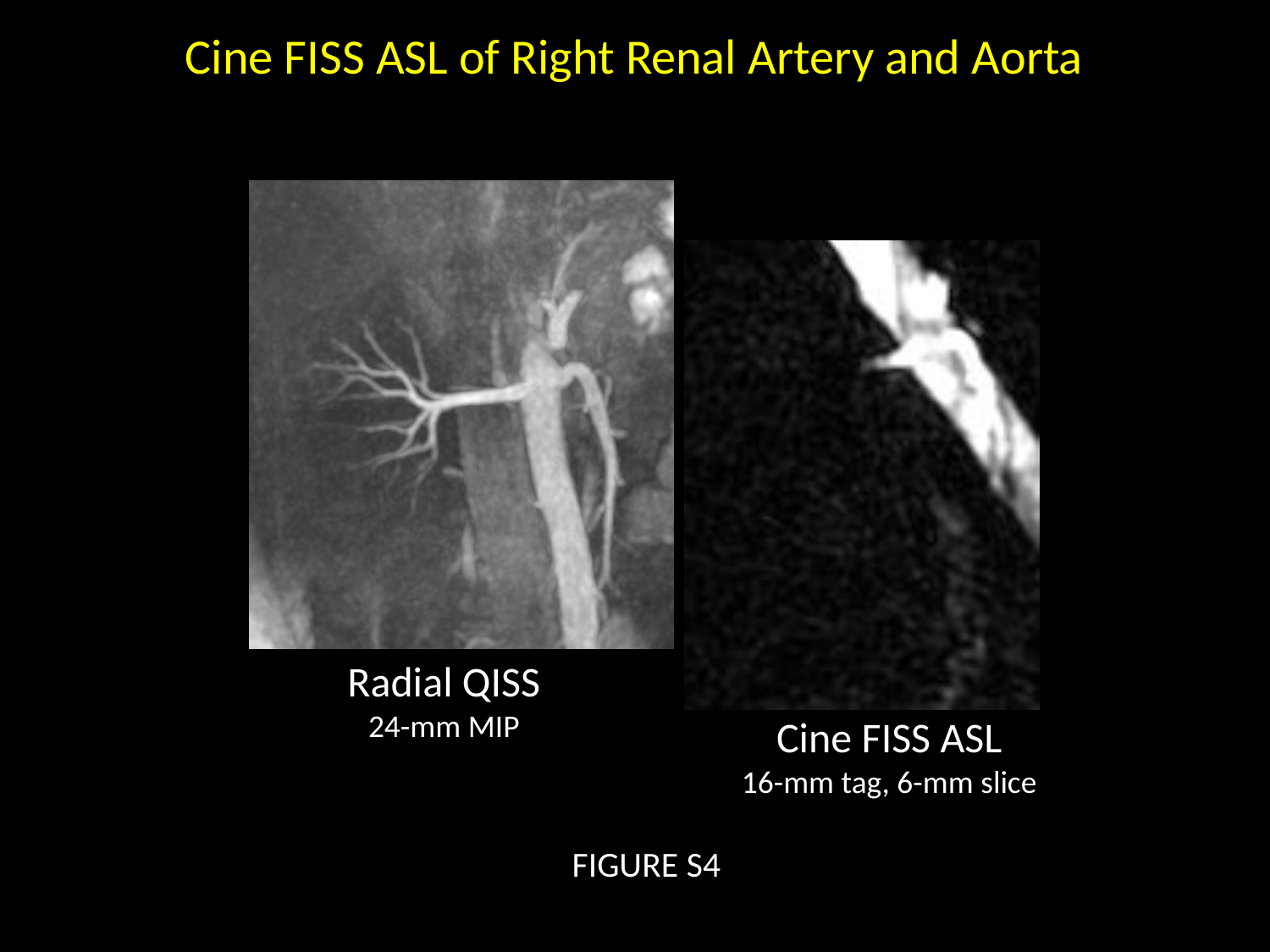

Cine FISS ASL of Right Renal Artery and Aorta
Radial QISS
24-mm MIP
Cine FISS ASL
16-mm tag, 6-mm slice
FIGURE S4

## Slide 5
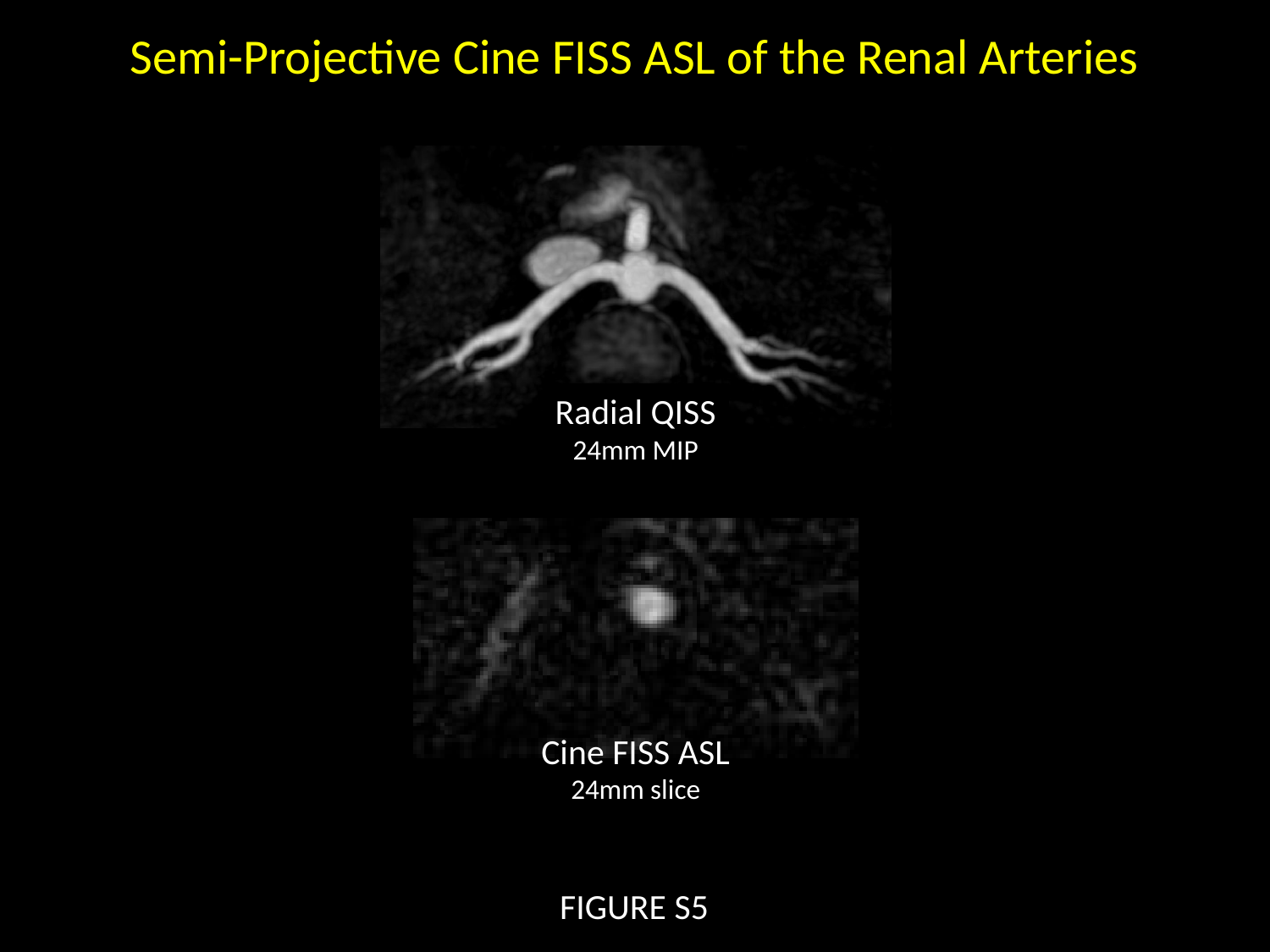

Semi-Projective Cine FISS ASL of the Renal Arteries
Radial QISS
24mm MIP
Cine FISS ASL
24mm slice
FIGURE S5

## Slide 6
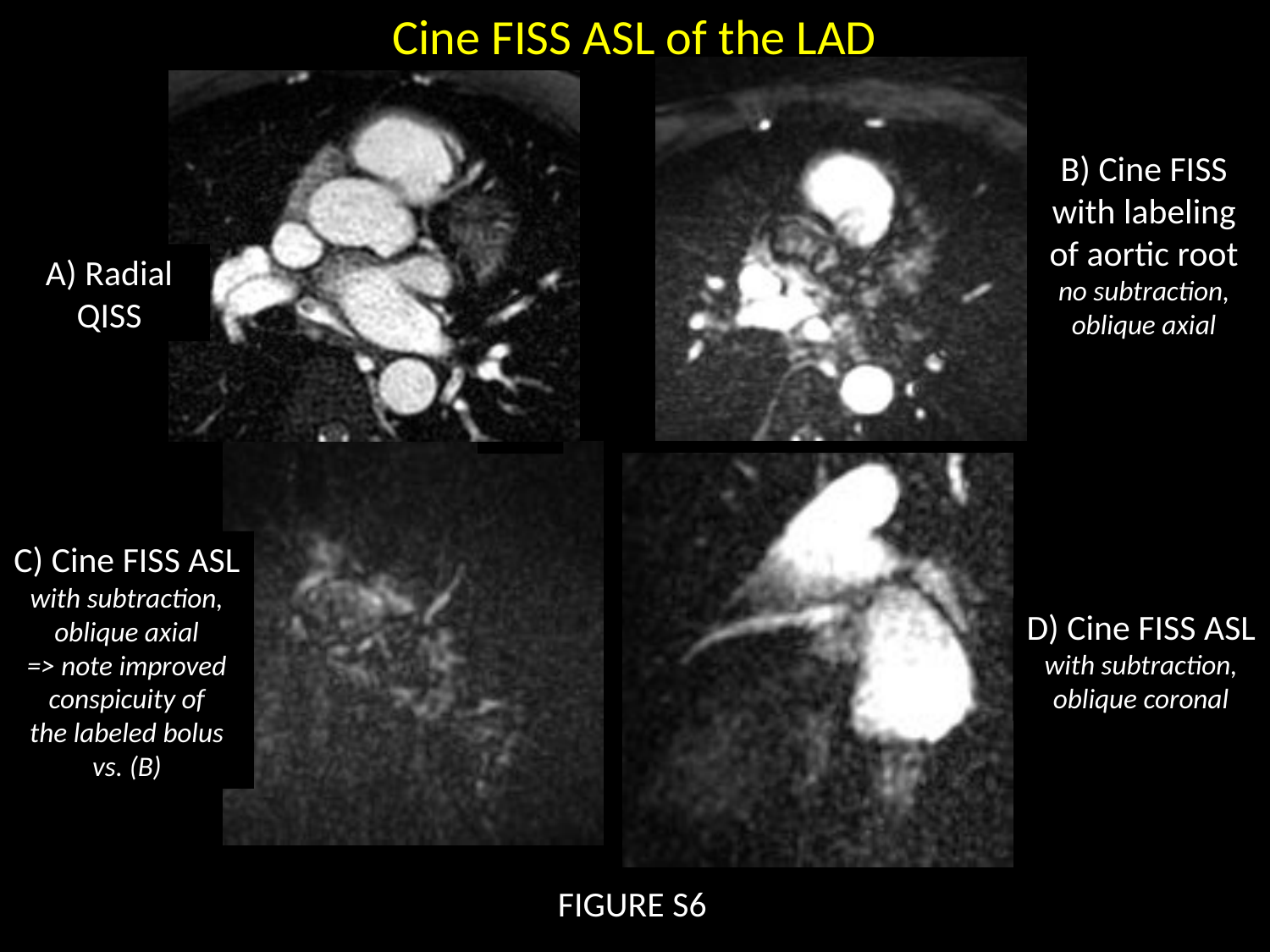

Cine FISS ASL of the LAD
B) Cine FISS with labeling of aortic root
no subtraction,
oblique axial
A) Radial QISS
Cine
FISS
C) Cine FISS ASL
with subtraction, oblique axial
=> note improved conspicuity of
the labeled bolus
vs. (B)
D) Cine FISS ASL
with subtraction, oblique coronal
FIGURE S6

## Slide 7
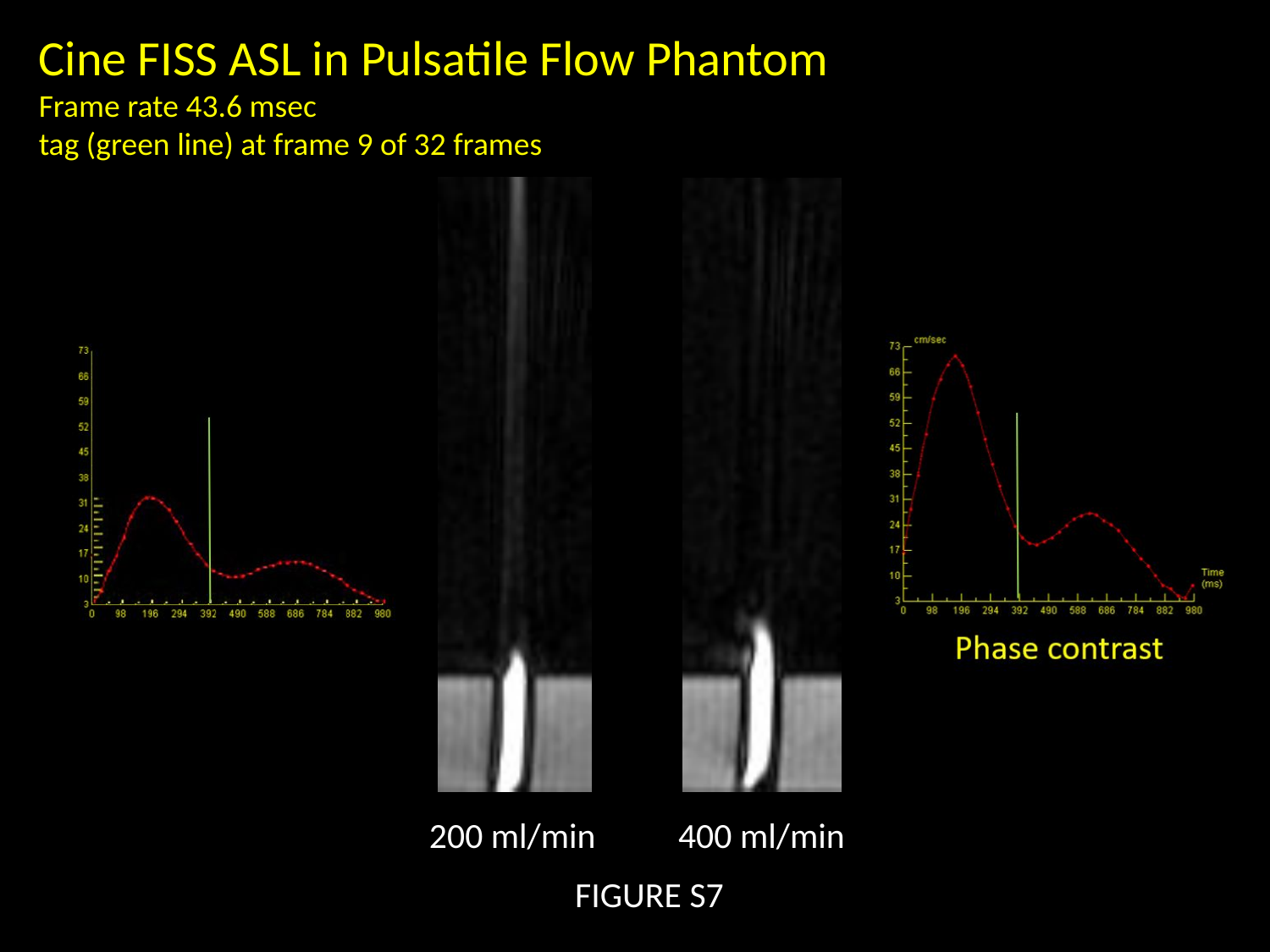

Cine FISS ASL in Pulsatile Flow Phantom
Frame rate 43.6 msec
tag (green line) at frame 9 of 32 frames
200 ml/min
400 ml/min
FIGURE S7

## Slide 8
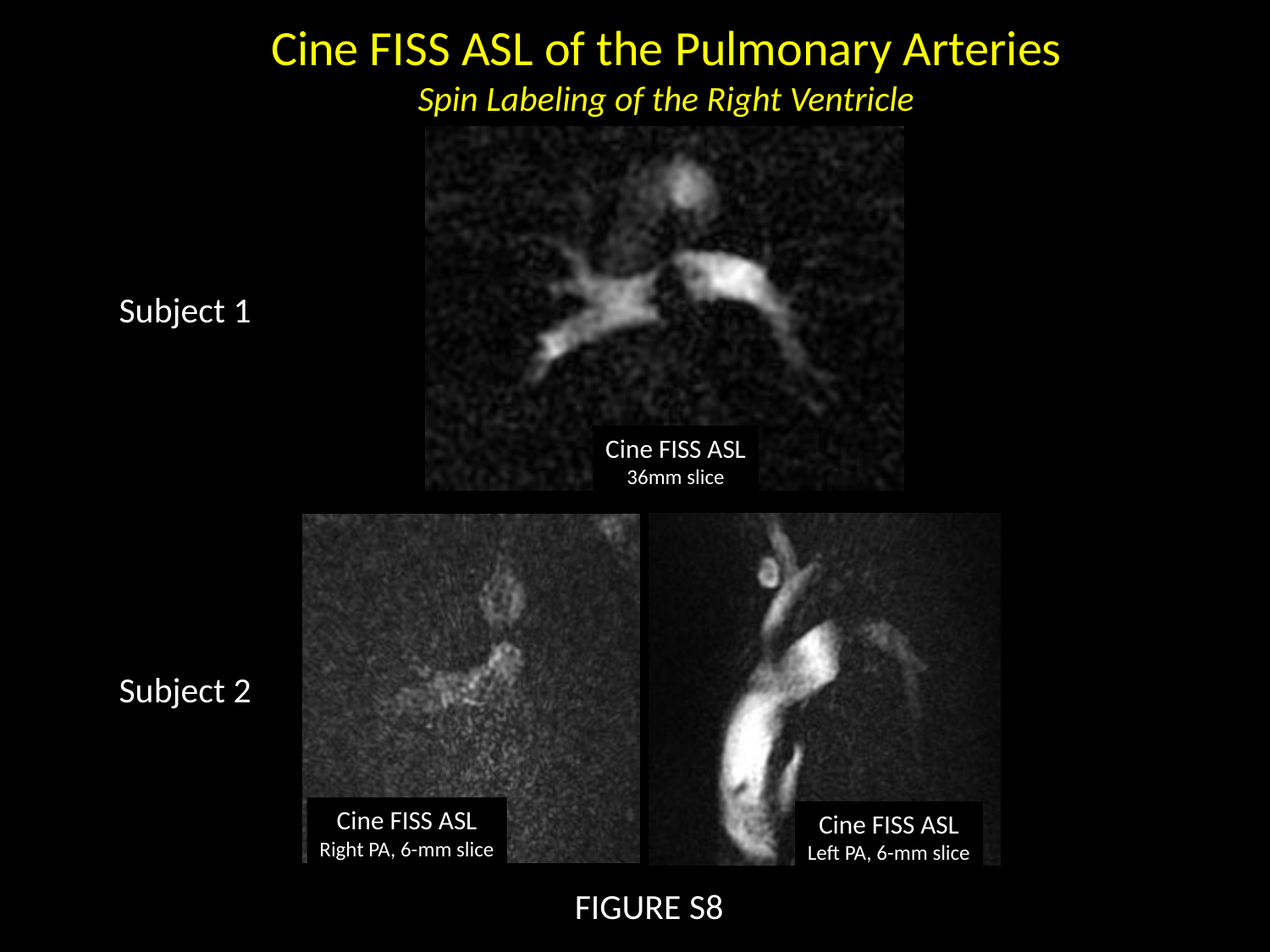

Cine FISS ASL of the Pulmonary Arteries
Spin Labeling of the Right Ventricle
Subject 1
Cine FISS ASL
36mm slice
Subject 2
Cine FISS ASL
Right PA, 6-mm slice
Cine FISS ASL
Left PA, 6-mm slice
FIGURE S8
